# P-629. The Health and Economic Impact of the PCV15 and PCV20 Priming Series during the First Year of Life in the US

**DOI:** 10.1093/ofid/ofae631.827

**Published:** 2025-01-29

**Authors:** Mark Rozenbaum, Benjamin Althouse, Maria J Tort, Alejandro D Cane, Raymond Farkouh

**Affiliations:** Pfizer Inc., Randstad, Noord-Holland, Netherlands; Pfizer, New York, New York; Pfizer Inc, Collegeville, Pennsylvania; Pfizer, New York, New York; Pfizer, Inc., Collegeville, Pennsylvania

## Abstract

**Background:**

Two pneumococcal conjugate vaccines, 15- (PCV15) and 20- (PCV20) valent formulations, are currently recommended for US infants in a 3+1 schedule. The first 3 doses are administered during the first year of life (‘priming doses’ at 2, 4, and 6 months) while the booster dose is given at 12 to 15 months. This study evaluated the health and economic impact of priming doses within the first year of life.

**Methods:**

Using a decision-analytic model, we calculated the health outcomes and economic implications from a healthcare payer perspective over a 12-month duration. Our epidemiological data were drawn from peer-reviewed studies. Estimates for vaccine effectiveness on invasive pneumococcal disease (IPD), inpatient and outpatient community-acquired pneumonia (CAP), and otitis media (OM) were extrapolated from established PCV13 effectiveness and PCV7 efficacy studies and adjusted for current serotype coverage of PCV15 and PCV20. Direct medical costs related to the treatment of IPD, CAP and OM were extracted from the literature and inflated to 2024 dollars. Aligned with CDC assumptions, we projected that children would experience 75.6% of the full vaccine effectiveness in their first year of life.

**Results:**

Vaccination of newborns in the United States at 2, 4, and 6 months using PCV20 could potentially prevent an estimated 137 cases of IIPD, 3,150 cases of pneumonia, 54,000 cases of OM, and 38 deaths during the first year of life. This could result in savings of approximately 76M dollars in direct medical costs. When compared to universal administration of PCV15, PCV20 offers greater case prevention due to its broader serotype coverage. Specifically, using PCV15 instead of PCV20 would result in 54 fewer prevented cases of IPD, 26,200 fewer cases of otitis media, 1,526 fewer pneumonia cases, and 16 fewer prevented deaths.

**Conclusion:**

This study showed that, in the US, vaccinating with PCV20 would yield a significantly greater health and economic return during the first 12 months of life compared to PCV15 due to the 5 additional serotypes covered by PCV20.

**Disclosures:**

**Mark Rozenbaum, PhD, M.B.A.**, Pfizer: Stocks/Bonds (Public Company) **Benjamin Althouse, PhD**, Pfizer: Stocks/Bonds (Public Company) **Maria J. Tort, PhD**, Pfizer Inc: Stocks/Bonds (Public Company) **Alejandro D. Cane, MD, PhD**, Pfizer: Employee|Pfizer: Stocks/Bonds (Public Company)|Pfizer: Stocks/Bonds (Public Company) **Raymond Farkouh, PhD**, Pfizer: Stocks/Bonds (Public Company)

